# Active Video Gaming Using an Adapted Gaming Mat in Youth and Adults With Physical Disabilities: Observational Study

**DOI:** 10.2196/30672

**Published:** 2021-08-26

**Authors:** Laurie A Malone, Ganisher K Davlyatov, Sangeetha Padalabalanarayanan, Mohanraj Thirumalai

**Affiliations:** 1 University of Alabama at Birmingham/Lakeshore Research Collaborative School of Health Professions University of Alabama at Birmingham Birmingham, AL United States; 2 Department of Health Administration and Policy Hudson College of Public Health University of Oklahoma Health Sciences Center Oklahoma City, OK United States; 3 Department of Health Services Administration University of Alabama at Birmingham Birmingham, AL United States

**Keywords:** exergaming, video games, disability, exercise, physical activity, enjoyment, dance mat, serious games, gaming mat, mobility impairment, physical impairment

## Abstract

**Background:**

A common leisure-time activity amongst youth and adults in the United States is video gameplay. Playing video games is typically a sedentary endeavor; however, to encourage an increased level of physical activity in an engaging and enjoyable way, active video gaming has become popular. Unfortunately, the accessibility of gaming controllers is often an issue for persons with disabilities. A commercial off-the-shelf (OTS) gaming mat was adapted to facilitate use by individuals with mobility impairments to address this issue.

**Objective:**

Our study aimed to examine energy expenditure, enjoyment, and gameplay experience in youth and adults with mobility impairment during active video gaming using an OTS and adapted versions of a gaming mat.

**Methods:**

The study used an observational design. During visit 1, physical function was assessed, and participants were given a familiarization period with the gaming system. For visit 2, based on observation during the physical function tests and discussion with the participant, it was decided whether the participant would play in a standing or seated position. For standing gameplay, the mat was placed on the floor, and for seated play, the mat was placed on a height-adjustable and tilt-adjustable tabletop. Metabolic data were collected during a 20-minute baseline and four 10-minute bouts of Wii Fit Plus gameplay, with 2 bouts on each of the mats (adapted and OTS). During gameplay, the research staff observed and rated participants’ ability to use the game controller (mat) and the quality of gameplay. At the end of each game set, participants reported their rating of perceived exertion on a scale from 0 to 10. During rest, participants completed the physical activity enjoyment scale. Participants also answered additional questions regarding the system's usability with each controller (adapted mat and OTS mat). Statistical analyses were computed using Stata 16 (version 16.1; StataCorp). Linear mixed-effects maximum likelihood regression was performed separately for individuals who could play standing and for those who played seated.

**Results:**

A convenience sample of 78 individuals with mobility impairments between the ages of 12 and 60 years (mean 39.6, SD 15.8) participated in the study. Of the sample, 48 participants played the video games in a seated position, while 30 played the games standing. Energy expenditure and heart rate tended to be higher in the OTS mat condition for seated players, while values were similar for both conditions among standing players. However, seated participants reported greater gameplay experience, and both groups exhibited a higher quality of gameplay during the adapted mat condition.

**Conclusions:**

Active video gaming using an adapted gaming mat provided an enjoyable exercise activity for individuals with mobility impairments. The use of the adapted controller provides a means by which this population can engage in light to moderate intensity active video gaming, thereby reducing sedentary leisure time.

**Trial Registration:**

ClinicalTrials.gov NCT02994199; https://clinicaltrials.gov/ct2/show/NCT02994199

## Introduction

A common leisure-time activity amongst youth and adults in the United States is video, with 227 million video gamers across the United States [1]. Recent statistics compiled by the Entertainment Software Association report that 74% of all households have at least one member who plays video games, with 67% of all adults and 76% of youth in the United States engaging in regular video gameplay [1]. Of those playing video games, 80% are adults, with an average age of 31 years. Across all players in the United States, 55% are male, and 45% are female, across a diverse population including those with disabilities [1]. Of note, the video game industry generates over $90 billion in annual economic output in the United States [2].

Stereotypical video gameplay conjures up images of hours spent fixated on a screen in sedentary repose. However, the scope of video gaming has broadened and been shown to provide a role in physical rehabilitation, a tool for health promotion and behavior change, and a means for improving mental health [3-8]. A specific genre of gameplay that moves beyond the sedentary is known as active video gaming (AVG) or exergaming. Such games require large body movements of the arms or legs as opposed to the simple finger motions for standard controller operation. The goal of AVG is to encourage an increased level of physical activity in an engaging and enjoyable way.

With fewer opportunities for exercise and countless barriers to participation in leisure-time physical activity (LTPA), people with disabilities experience significantly higher levels of sedentary behavior, physical inactivity, and associated health risks [9,10]. Contributing factors include issues with transportation and facility access, costs associated with specialized equipment (eg, sports wheelchair), the absence of staff trained to accommodate special needs, and boredom with the limited options available [11-18]. Replacing sedentary behaviors with AVG play holds promise as a way to reduce those barriers and increase LPTA in people with disabilities [19-23]. Moreover, AVGs have been described as having the potential to be an engaging introduction to physical activity. Such games open the door to interest and participation in other forms of physical activity for persons with disabilities [21].

Of those who play video games in the United States, 73% own a game console [1]. However, accessibility issues with most gaming consoles are a barrier for many with mobility limitations [20,21]. For instance, many hand controllers are difficult for those with upper extremity impairments to hold and manipulate for gameplay. Likewise, individuals who use a wheelchair are unable to play Wii Fit games using the OTS Wii board. Others have worked on gaming interface adaptations to allow people with disabilities to play video games [24-27]; however, limited research and development efforts have focused on improving the accessibility of commercially available gaming controllers for use with AVGs. Our previous work as part of the Rehabilitation Engineering Research Center on Interactive Exercise Technologies and Exercise Physiology for People with Disabilities at the University of Alabama at Birmingham/Lakeshore Foundation Research Collaborative examined the accessibility of commercial off-the-shelf video game controllers, including the Wii Fit balance board system and dance pad gaming mats. Data on gameplay, participants’ ability to use the controllers, user feedback, and research staff qualitative observations indicated that both controllers needed adaptation to overcome certain deficiencies for successful gameplay. These data were fed to the engineering team to develop an adapted gaming balance board and an adapted gaming mat. Subsequent research demonstrated that the adapted gaming board could increase accessibility, provide physical activity, and allow enjoyable gameplay action for people with mobility impairments [28-30]. The development of adapted video game controllers offers an innovative approach to overcoming various barriers to exercise in people with disabilities.

The aim of the study was to examine energy expenditure via metabolic equivalents (METs) and enjoyment, as measured by the physical activity enjoyment scale (PACES), in individuals with physical disabilities, specifically those with mobility impairments (ie, unable to stand, balance issues, poor motor control, or unable to use lower extremity for gameplay), during AVG play using an OTS and adapted versions of a gaming mat. Also of interest were differences in heart rate, rating of perceived exertion (RPE), quality of gameplay, and gameplay experience between the two gaming mats.

## Methods

### Design and Setting

This was an observational study conducted at Lakeshore Foundation, a community fitness center in the southeastern United States that provides specialized physical activity, sport, and recreation opportunities for individuals with physical disabilities and chronic health conditions. The University’s Institutional Review Board for Human Use approved all study procedures (IRB-150909002).

### Participants

Eligibility criteria included 10 to 60 years of age, a confirmed diagnosis of lower extremity mobility limitation (eg, spina bifida, cerebral palsy, muscular dystrophy, 1 year following a spinal cord injury, multiple sclerosis, stroke, or limb loss) with partial or full use of upper extremities and use of an assistive device (eg, cane, walker, or wheelchair) or problems with gait, balance, or coordination. In addition, participants were excluded if they had an unstable cardiovascular condition, a visual impairment that interfered with playing video games, or weighed over 350 lbs (159 kg), including their assistive device.

### Procedures

All testing for the research study took place in the Exercise and Sport Science Laboratory at Lakeshore Foundation. For this study, participants came to the lab 3 times, generally within a 3-week period. The laboratory housed a station for AVG play equipped with a 58-inch Sony high-definition television, gaming console, and controllers (gaming mats).

Before assessments began, each participant was fitted with a heart rate monitor (S610; Polar). Heart rate (beats per minute) was recorded every 3 to 4 seconds for the duration of gameplay. To assess the participants' physical function, 18 activities from the International Classification of Functioning, Disability and Health (ICF) [31,32] were preselected for use. Participants were asked to perform these activities during the first session. The research staff selected a number (0-4) to reflect the level of difficulty the participant had completing each task. As defined in the ICF manual, the scoring was as follows: 0=“no difficulty,” 1=“mild difficulty,” 2=“moderate difficulty,” 3=“severe difficulty,” and 4=“complete difficulty.” The specific ICF tasks selected for use in this study were based on a consensus among the research staff as to which mobility activities listed in the ICF had the potential to be required for AVG play (eg, standing, reaching, throwing, and jumping) based on prior observations. Scores on each of the 18 tasks were added together as a composite to represent participants’ physical function. A lower physical function score indicated greater functional ability on the selected tasks. A description of the specific tasks has previously been published [28].

In addition, the participants rated their physical function using the PROMIS SF v1.0—physical function 20a measure. This self-reported measure assesses an individual’s perception of their current function, specifically the upper and lower extremities and central region (trunk and neck). It also evaluates their ability to complete instrumental activities of daily living (eg, running errands). A single physical function capability score is obtained from the measure [33].

### Adapted Gaming Mat

Prior work by our exercise science and engineering teams had identified deficiencies in commercial OTS gaming mats that could be improved upon to enhance accessibility. The deficiencies were based on the assumption that many players with mobility impairments would need to play while seated with the mat placed on a tabletop in front of them. The specific issues identified were:

1. Large area button layout: The original OTS button layout was designed to accommodate players who could stand and exert body weight force through the lower extremities, utilizing a large 3 ft x 3 ft playing surface, over which the 8 controller buttons and 2 menu buttons were distributed. This large span of the buttons made it difficult for a seated player to reach all the buttons.
2. High button actuation force: The buttons were designed for high actuation force as would be common when used by a standing player stepping or stomping with their feet, but becomes difficult for use with the hands.
3. “Dead” spots in button area: The underlying design of the mat buttons was such that “dead” spots existed within the area of each button when trying to depress the button with a couple of fingers rather than a whole foot.

**Figure 1:**
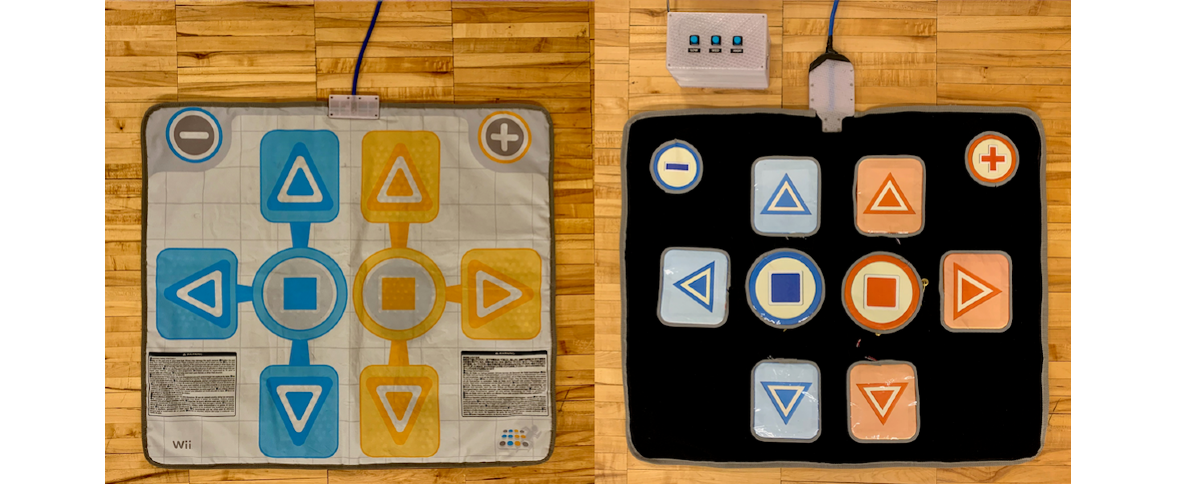
Adapted foot gaming mat (left) and adapted hand (right) mat. Note, the exterior shell of the foot mat looks identical to an off-the-shelf mat, given that only the inside components were re-engineered.

The OTS mat (2007; Wii) was re-engineered to create an adapted mat design to address the issues described above. Two adapted mats were built, one specifically for standing players to play with their feet and another for seated players to play with their hands (Figure 1). For both adapted mats, the underlying button technology was replaced to increase the level of accessibility. Both mats included variable button actuation force (sensitivity adjustment) and consistent button response over the entire button area. A reconfigurable button layout design using hook and loop attachments was also incorporated for the mat to be used by seated players.

### Active Video Gameplay

Upon arrival at the lab, participants pulled a number to determine which version of the controller they would play first (adapted or OTS), then the participant would also draw a number to determine which game set (explorer or outdoor challenge) they would play first (Textbox 1). Whichever game set was selected first would be played on both versions of the mat (adapted and OTS), and then the second game set would be played on each version. Based on observations during the physical function tests and discussions with the participant, it was decided whether the participant would play in a standing or seated position. For standing play, the mat was placed on the floor, and for seated play, the mat was placed on a height-adjustable and tilt-adjustable tabletop. Participants were then set up with the portable metabolic system (COSMED K4b2) and a heart rate monitor to assess pulmonary gas exchange and indirect calorimetry. Data collection began with a 20-minute rest period to measure the resting energy expenditure. For the rest period, participants sat quietly with no speaking or distractions besides lightly reading a magazine or viewing their cellular phone. Next, gameplay began with continuous gas exchange and heart rate data collection. Gameplay consisted of four 10-minute sets with a rest period of 5 minutes after each game set.

**Mini-games played on both off-the-shelf (OTS) and adapted gaming mat.**

**Active life explorer**

- **Crocodile stomper:** Step on the crocodiles approaching from all sides to drive them away.
- **Airplane panic:** Save an airplane from a bandit and fly back to safety.
- **Kraken battle:** Fight a sea monster in an attempt to survive.
- **Mummy’s tomb:** Run away from the mummies, lock the gates, and escape the tomb.
- **Jungle vine ruins:** Run, jump, and climb through the jungle ruins to get the treasure.

**Active life outdoor challenge**

- **Sprint challenge:** Run along the straight course as fast as possible.
- **Jump rope:** Jump over the rope following the rhythm, becoming faster as time progresses.
- **Conveyor runner:** Run along the moving conveyor and jump over obstacles aiming to stay on as long as possible.
- **Log leaper:** Jump to avoid the oncoming logs and stay on the platform as long as possible.
- **Head-on hurdler:** Run and jump over the hurdles placed along the course.
- **Mole stomper:** Whack the moles popping up from different holes on all sides.

Standing players used their feet to run, jump and stomp on the required mat buttons following the on-screen prompts; seated players used their hands.

During gameplay, research staff observed and rated the ability to use the game controller (mat) and the quality of gameplay. The ability to use the game controller assessed the participant’s difficulty or ease of using the controller as required for the game and was rated on a scale of 1-5 (1=extreme difficulty, 2=severe difficulty, 3=moderate difficulty, 4=mild difficulty, and 5=no difficulty). To assess gameplay quality, the research staff considered the participants’ degree of general game manipulation and user actions as prompted by the game compared to how a gamer without a physical disability would play as observed during testing with staff and preliminary usability testing. Quality of gameplay ranged on a scale from 1-5 (1=poor, 2=fair, 3=moderate, 4=good, and 5=excellent). Two research staff worked together for all testing sessions. Both recorded scores for quality of gameplay and controller usage and came to a consensus for the final scores at the end of the session. All sessions were videotaped, so in the event that testers could not come to a consensus, the recording would be available for review. Staff observation scores were combined for the 2 games.

At the end of each game set, participants reported their rating of perceived exertion on a scale from 0-10, with 0=not tired at all and 10=very, very tired. During rest periods, participants completed a feedback survey that included PACES [34]. The PACES includes 16 statements such as “I enjoyed it,” “It was very exciting,” “I felt bored,” and “It was no fun at all.” All items were rated by the participant on a 5-point scale ranging from 1=strongly disagree to 5=strongly agree. After reverse scoring 7 items, a final score was computed by calculating the average of the 16 items.

Participants also answered additional questions regarding the usability of the system with each controller (adapted mat and OTS mat): “How hard was it for you to use the gaming mat?” (unable to use=1 to very easy= 5); “Do you feel like you were able to successfully play the game?” (strongly disagree=1 to strongly agree=5); and “Did you feel like the mat recognized your body movements?” (strongly disagree=1 to strongly agree=5). Participant scores were combined for the 2 games. To gauge participants’ perspective of the gameplay experience and any differences between the two games, they were asked, “Do you consider playing this game as an exercise, fun activity, both, or neither?”

### Data Analysis

As the sample size was small, the data did not meet normality assumptions. Therefore, Wilcoxon signed-rank nonparametric tests were run to identify the difference between the use of the 2 gaming mats (OTS vs adapted). Given the variation in gameplay style between seated (mat on the table) and standing (mat on the floor), all the tests were conducted separately for sitting and standing participants. As the same person played games using the OTS and adapted gaming mats, measurements cannot be considered independent. To control for within-subject variability and accommodate missing data, linear mixed-effects maximum likelihood regression tests were conducted to determine any statistically significant differences between the means of variables when participants used OTS versus adapted mats. A significance level of .05 was used in evaluating the statistical tests. Data management and analyses were performed using Stata 16 (version 16.1; StataCorp).

## Results

A convenience sample of 78 individuals with mobility impairments between the ages of 12 and 60 years (mean 39.6, SD 15.8) volunteered for the study. The sample included 34 females and 44 males, with 39 non-Hispanic Whites, 37 Blacks, 1 Hispanic, and 1 Other. Of the sample, 48 participants played the video games in a seated position, while 30 played the games standing.

**Figure 2:**
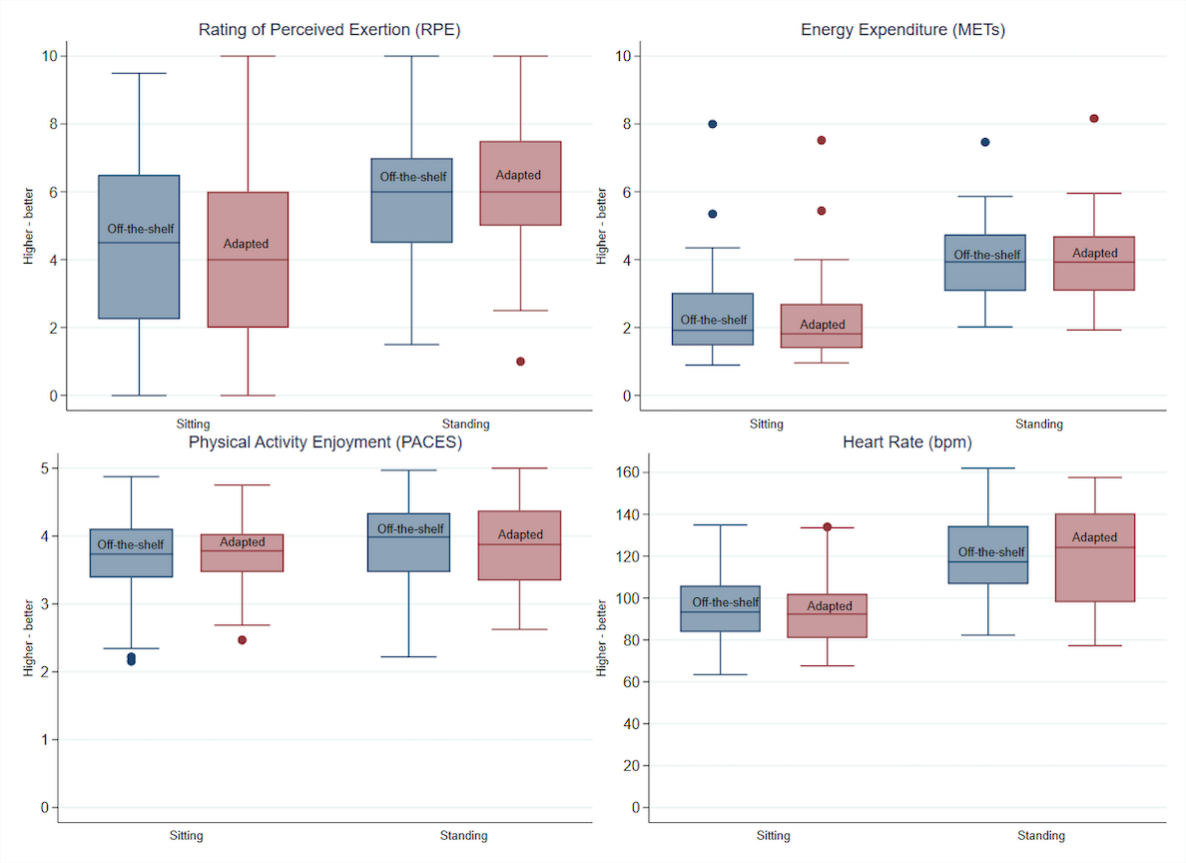
Descriptive statistics of active video gaming (AVG) gameplay using off-the-shelf (OTS) vs adapted gaming mat. bpm: beats per minute; MET: metabolic equivalent; PACES: physical activity enjoyment scale; RPE: rating of perceived exertion.

**Figure 3:**
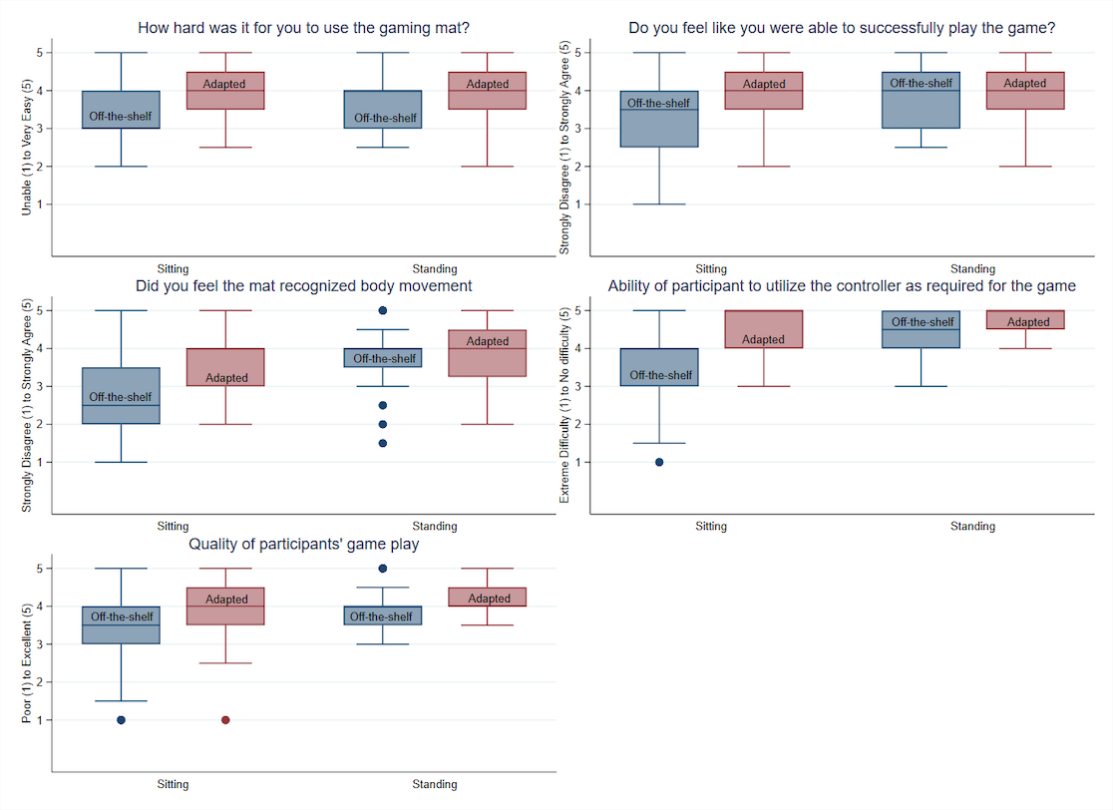
Gameplay experience of participants and staff observations for off-the-shelf (OTS) versus adapted gaming mat.

Descriptive statistics (Table 1) present the differences in a series of variables captured during gameplay by participants while using the OTS mat versus the adapted gaming mat. The Wilcoxon signed-rank test results are displayed with the medians and interquartile ranges for all outcome measures. There were significant differences, including gameplay METs and heart rate for sitting participants where values were higher during the OTS mat condition compared to play using the adapted mat. However, for seated players, all self-reported ratings of the gameplay experience and staff observations of gameplay were significantly higher for the adapted mat in comparison to the OTS mat. For standing players, staff observations of the ability to use the gaming mat were significantly higher for the adapted mat compared to the OTS mat. Box plots are also used to display the results in Figures 2 and 3.

Mixed-effect model results are reported in Table 2. In all models, participants’ age and performance were used as control variables. Among the participants that played seated, lower energy expenditure (*β*=–.18; *P*<.001) and lower heart rate (*β*=–2.09; *P*=.01) were reported when they used the adapted mat compared to the OTS mat. For instance, seated participants using the OTS mat had a 2 point increase in heart rate in comparison to gameplay using the adapted mat.

Adapted mat use, however, was associated with higher scores in participants’ measures of the gameplay experience and staff observations of controller use and quality of gameplay. For example, sitting participants using the adapted mat scored 0.68 units higher in ease of using the gaming mat, 0.62 units higher in their perceived ability to successfully play the game, 0.85 units higher in their perception that the gaming mat effectively recognized body movement. In addition, staff observations were 0.98 units higher in rating the participants’ ability to use the adapted controller and 0.55 units higher in the quality of gameplay compared to their observations during gameplay with the OTS mat. Likewise, for participants that played the games standing, staff reported higher scores in their ability to use the controller (*β*=.35; *P*<.001) and quality of gameplay (*β*=.26; *P*=.001) with use of the adapted mat. Participant responses regarding each game as fun or exercise were tallied. For each game and both conditions (sitting and standing), the majority (66%-83%) of responses indicated the activity as both exercise and fun.

**Table 1:** Descriptive statistics for gameplay variables, participant ratings, and staff observations (N=78).

Variables				OTS mat		Adapted mat		*P* value
**RPE (0-10), median (IQR)**								
		Sitting	4.5 (2.3-6.5)		4.0 (2.0-6.0)		.11	
		Standing	6.0 (4.5-7.0)		6.0 (5.0-7.5)		.52	
**Enjoyment (PACES^a^), median (IQR)**								
		Sitting	3.7 (3.4-4.1)		3.8 (3.5-4.0)		.37	
		Standing	4.0 (3.5-4.3)		3.9 (3.3-4.4)		.11	
**Energy Expenditure (METs^b^), median (IQR)**								
		Sitting	1.9 (1.5-3.0)		1.8 (1.4-2.7)		<.001	
		Standing	3.9 (3.1-4.7)		3.9 (3.1-4.7)		.21	
**Heart rate (bpm^c^), median (IQR)**								
		Sitting	93.3 (83.8-105.9)		92.3 (80.9-102.1)		.001	
		Standing	117.3 (106.7-134.5)		124.1 (98.0-140.4)		.32	
**Participant ratings**								
	**How hard to use gaming mat (1=unable to 5=very easy), median (IQR)**							
		Sitting	3.0 (3.0-4.0)		4.0 (3.5-4.5)		<.001	
		Standing	4.0 (3.0-4.0)		4.0 (3.5-4.5)		.11	
	**Able to successfully play (1=strongly disagree to 5=strongly agree), median (IQR)**							
		Sitting	3.5 (2.5-4.0)		4.0 (3.5-4.5)		<.001	
		Standing	4.0 (3.0-4.5)		4.0 (3.5-4.5)		.24	
	**Mat recognized body movement (1=strongly disagree to 5=strongly agree), median (IQR)**							
		Sitting	2.5 (2.0-3.5)		4.0 (3.0-4.0)		<.001	
		Standing	4.0 (3.5-4.0)		4.0 (3.3-4.5)		.41	
**Staff Observations**								
	**Ability to use gaming mat (1=extreme difficulty to 5=no difficulty), median (IQR)**							
		Sitting	4.0 (3.0-4.0)		5.0 (4.0-5.0)		<.001	
		Standing	4.5 (4.0-5.0)		5.0 (4.5-5.0)		<.001	
	**Quality of gameplay (1=poor to 5=excellent), median (IQR)**							
		Sitting	3.5 (3.0-4.0)		4.0 (3.5-4.5)		<.001	
		Standing	4.0 (3.5-4.0)		4.0 (4.0-4.5)		.004	

^a^PACES: physical activity enjoyment scale.

^b^MET: metabolic equivalent.

^c^bpm: beats per minute.

**Table 2:** Mixed-effects model illustrating gameplay variables, participant ratings, and staff observations in using off-the-shelf (OTS) versus the adapted mat.^a^

Variables		Adapted (vs OTS) coefficient		*P* value
**RPE^b^**				
	Sitting	–0.38	.15	
	Standing	0.23	.34	
**Enjoyment**				
	Sitting	0.05	.42	
	Standing	–0.09	.09	
**Energy Expenditure (METs^c^)**				
	Sitting	–0.18	<.001	
	Standing	–1.10	.18	
**Heart rate (bpm^d^)**				
	Sitting	–2.09	.01	
	Standing	–1.08	.41	
**Difficulty using the gaming mat**				
	Sitting	0.68	<.001	
	Standing	0.20	.10	
**Able to successfully play**				
	Sitting	0.62	<.001	
	Standing	0.08	.48	
**Mat recognized body movement**				
	Sitting	0.85	<.001	
	Standing	0.21	.30	
**Ability to use gaming mat**				
	Sitting	0.98	<.001	
	Standing	0.35	<.001	
**Quality of gameplay**				
	Sitting	0.55	<.001	
	Standing	0.26	.001	

^a^Control variables: age and self-reported physical function score.

^b^RPE: rating of perceived exertion.

^c^MET: metabolic equivalent.

^d^bpm: beats per minute.

## Discussion

### Principal Findings

The benefits of regular physical activity are clear, and the physical activity guidelines to achieve these benefits apply to everyone [35]. However, only approximately 20% of adults and youth in the United States are sufficiently active to improve health [36]. As noted in the Physical Activity Guidelines for Americans [35], any amount of physical activity has some health benefits. However, individuals with disabilities have fewer opportunities to engage in LTPA, thereby having even higher physical inactivity rates [9] and at greater risk for chronic disease. In the general population, AVG has been shown to be a fun and engaging way to increase physical activity [37,38].

Increases in energy expenditure during AVG have been reported for individuals with mobility impairments. For example, among children and adults with cerebral palsy moderate levels of physical activity were achieved during Wii Sports gameplay while standing [39,40] and seated [22]. Similar results were found for adults poststroke who played Wii Sports in a standing position [41]. In another study, moderate-intensity activity was achieved in both seated and standing positions among poststroke adults playing Xbox Kinect and Wii games [42]. Increases in energy expenditure were also found in seated players with spinal cord injury during Wii Sports and PlayStation 3 [43,44]. Other efforts have focused on supplementing existing exercise equipment with gaming for various youth and adult populations (ie, spina bifida, spinal cord injury, and cerebral palsy), achieving increases in exercise intensity [24] and enjoyment [45-47].

Incorporating accessibility features into the design and development of AVG controllers has the potential to reduce barriers to physical activity and improve the overall gameplay experience. However, little work has been done to incorporate design features into commercially available AVG controllers as a way to engage and promote active play in persons who require a wheelchair for mobility or need to play in a seated position. A feasibility study using an adapted upper-extremity Dance, Dance Revolution gaming mat on a table found increases in energy expenditure among 3 nonambulatory functionally diverse young adults [22]. Recognizing the potential for AVG play to increase energy expenditure among people with mobility impairments led our team to develop 2 adapted gaming controllers, an adapted version of the Wii Fit balance board [28,29] and the Wii gaming mat as described in this study. Previously reported results of the adapted Wii board study found that participants were able to achieve light-intensity (<3 METs) to moderate-intensity (3-6 METs) exercise with high levels of enjoyment [30]. The current study aimed to examine similar outcomes in persons with mobility impairments using OTS and adapted versions of the Wii gaming mat during AVG play.

Use of the adapted gaming mat in this study allowed for successful AVG play by a cohort of youth and adults with physical disabilities, specifically lower extremity mobility limitations. Furthermore, individuals were able to play either seated or standing, depending on their functional needs. The gameplay was enjoyable, and participants achieved light-intensity to moderate-intensity exercise. Although significant differences in energy expenditure and enjoyment between the 2 gaming mats were not found, the subjective experience of the seated players was improved by the adapted mat. As shown in the results, seated players reported greater ease of use, success, and recognition of body movements during gameplay on the adapted mat. Additional comments from the participants suggested a preference for the adapted mat due to it being more sensitive to their movement inputs (ie, hand or foot touch) and less frustrating. For the seated players, the adjustable button placement and smaller play area contributed to greater gameplay success by reducing the required reach and being more responsive. These features contributed to better control and players feeling more competitive in the games and achieving better scores. As noted, due to the increased sensitivity of the adapted mat, less force was required for game input. This feature may have inadvertently resulted in the slightly lower MET values for seated players during the adapted condition. However, with continued use and extended gameplay sessions, the requirement of less force would help minimize upper extremity injuries while having the potential to increase energy expenditure with greater familiarization, practice, and selection of games. Regardless, engagement in AVG play using the adapted mat will at minimum reduce sedentary time and produce light-intensity to moderate-intensity exercise for all players.

### Limitations

As an observational study, the generalizability of the results is limited, and claims regarding causality or efficacy cannot be made. Individuals were recruited from a community organization that provides physical activity, recreation, and sports programming for individuals with physical disabilities. Current physical activity levels varied among participants, as did their experience with video gaming. Measurements of exercise intensity (RPE, METs, and heart rate) may have been influenced by various factors not accounted for, such as the nature of the preselected games, the limited amount of time for participants to get familiarized with the gaming system, medications, and discomfort wearing the COSMED system for the measurement of oxygen consumption. Some degree of gameplay learning may have occurred during data collection, given the short familiarization period. In addition, since participants played only a select group of games, potential differences in enjoyment and energy expenditure between OTS and adapted controllers may not have been fully captured.

Furthermore, with regard to the assessment of exercise intensity based on METs, it must be noted that values do not consider the effect of impairment level on exercise intensity. Future studies should expand participant recruitment, conduct age group comparisons, examine a wider range of video games, provide an extended familiarization period, and compare outcomes during AVG play to other leisure-time physical activities. Further analyses with a larger sample that explore the multivariate correlation and variance-covariance matrices in the outcome measures (RPE, heart rate, MET, and PACES) would provide a better understanding of the relationship between exercise intensity and enjoyment and of clinically meaningful differences during AVG in this subpopulation.

### Conclusions

The adapted gaming mat allowed adults and youth with mobility impairments to engage in AVG, seated or standing. Energy expenditure and RPE indicated the activities to be light to moderate intensity. Although energy expenditure and heart rate tended to be higher in the OTS mat condition for seated players, the adapted mat provided the advantage of greater sensitivity, better responsiveness, and adjustable gameplay area. The adapted mat with adjustable buttons allowed for varying upper extremity mobility and arm reach lengths, thereby facilitating greater success and quality of gameplay. Overall, the adapted gaming mat provides an enjoyable option for increasing LTPA.

## Abbreviations

ACL: Administration for Community Living
AVG: active video gaming
HHS: Department of Health and Human Services
ICF: International Classification of Functioning, Disability and Health
LTPA: leisure-time physical activity
MET: metabolic equivalent
NIDILRR: National Institute on Disability, Independent Living, and Rehabilitation Research
OTS: off-the-shelf
PACES: physical activity enjoyment scale
RPE: rating of perceived exertion
